# Increased cardiovascular risk in Korean patients with systemic lupus erythematosus: a population-based cohort study

**DOI:** 10.1038/s41598-024-51546-1

**Published:** 2024-01-11

**Authors:** Jung-Yong Han, Soo-Kyung Cho, Hyoungyoung Kim, Yena Jeon, Gaeun Kang, Sun-Young Jung, Eun Jin Jang, Yoon-Kyoung Sung

**Affiliations:** 1 Department of Rheumatology, Hanyang University Hospital for Rheumatic Diseases, Seoul, Republic of Korea; 2 Hanyang University Institute for Rheumatology Research, Seoul, Republic of Korea; 3 Department of Statistics, Kyungpook National University, Daegu, Republic of Korea; 4 College of Pharmacy, Chung-Ang University, Seoul, Republic of Korea; 5 Department of Information Statistics, Andong National University, Andong, Republic of Korea

**Keywords:** Systemic lupus erythematosus, Cardiovascular diseases, Epidemiology

## Abstract

To determine the increased risk of major adverse cardiovascular events (MACE) in patients with systemic lupus erythematosus (SLE) compared to the general population in Korea. Using data from the National Health Insurance Service database spanning 2008 to 2018, incident SLE patients aged 18 years and above were selected along with a 1:4 age- and sex-matched control group. The crude incidence rate (IR) of MACE was calculated as the number of events per 1000 person-years and the IR ratio (IRR) for MACE was adjusted using generalized estimating equations. Subgroup analysis was conducted to evaluate the risk differences of overall MACE and its composites based on age and sex stratification. The study included 8568 SLE patients and 34,272 controls. The cumulative IR of MACE per 1000 person-years in SLE patients and controls were 4.08 and 1.30, respectively. After adjusting for confounders, SLE patients had a higher risk of MACE compared to the general population (adjusted IRR of 2.40 [95% confidence interval [CI] 1.88–3.05]), with no gender differences observed. The increased risk of MACE in SLE patients was highest in the 18–39 age group (IRR 11.70, 95% CI 5.95–23.01) and gradually decreased with age. The increased risk of ischemic stroke (IRR 2.41, 95% CI 1.84–3.15) and myocardial infarction (IRR 2.19, 95% CI 1.30–3.68) in SLE patients was comparable. The risk of MACE in SLE patients is 2.40 times higher than that of the general population, with a higher relative risk observed in younger individuals.

## Introduction

Systemic lupus erythematosus (SLE) is an autoimmune disorder characterized by a diverse range of conditions that can affect multiple organ systems, including the kidneys, gastrointestinal tract, and central nervous system^1–3^. Among the various complications associated with SLE, cardiovascular manifestations are prevalent and can present as pericarditis, valvular abnormalities, and coronary artery disease (CAD)^4^. The association between SLE and an increased frequency of CAD has been well-established^5^.

Cardiovascular events (CVE) are a leading cause of death in SLE patients, largely due to a high incidence rate of myocardial infarction (MI), stroke, and heart failure^6^. The risk of CVE in SLE patients has been documented to be three-to-four-fold higher than that of the general population^7^. And, another study revealed a five-fold higher risk of CVE in SLE patients, especially in females^8^. However, varying definitions of CVE have contributed to differences in reported results because CVE encompasses a wide range of cardiovascular conditions, including CAD, heart failure, stoke, and peripheral arterial disease^9^. In contrast, major adverse cardiovascular events (MACE), such as MI, stroke, or cardiac death, are usually used to specifically capture serious and potentially life-threatening cardiovascular diseases and serve as clinically significant measures for assessing cardiovascular risk^10^.

The impact of MACE on SLE patients is influenced by various factors, including age, gender, disease duration, and severity^11^. Racial disparities have also been identified as factors contributing to the development of MACE among SLE patients, with studies reporting a higher risk among Black individuals compared to White Americans, whereas Asians demonstrated a lower risk of MI compared to Whites^12^. Furthermore, population-based studies conducted in the United States and Korea have consistently demonstrated an increased risk of MACE, including MI and cardiac death, in SLE patients compared to the general population^13,14^. A longer disease duration was also associated with a higher risk of MACE in SLE patients^15^. However, the impact of age, gender, and time of diagnosis on MACE risk in Korean patients with SLE remains unclear.

Therefore, the objective of our study was to determine the risk of MACE in SLE patients compared to the general population and investigate the trends of MACE risk following SLE diagnosis in Korea.

## Materials and methods

### Data source and study population

The study utilized the Korean National Health Insurance Service (NHIS), which is a comprehensive healthcare coverage system in the Republic of Korea. The NHIS covers almost the entire population of the country, with only a small percentage supported by the Medical Aid program. The Korean National Health Insurance Database (NHID) contains a vast amount of health and medical information, including demographic data, medical claims, and prescription records, for individuals covered by the NHIS and the Medical Aid program^16^.

To identify patients with SLE, the NHID was used to identify individuals with both ICD-10 code (M32.0) and rare intractable disease (RID) code (V136) assigned to SLE. Our study included all prevalent SLE patients aged 18–79 years who were registered in the RID program between 2008 and 2018. These patients had to meet the strict criteria for a classification of SLE based on the 1997 Update of the 1982 American College of Rheumatology Revised Criteria^17,18^. The fulfillment of these criteria was carefully reviewed by physicians, as patients registered in this program received financial support from government aid.

**Figure 1:**
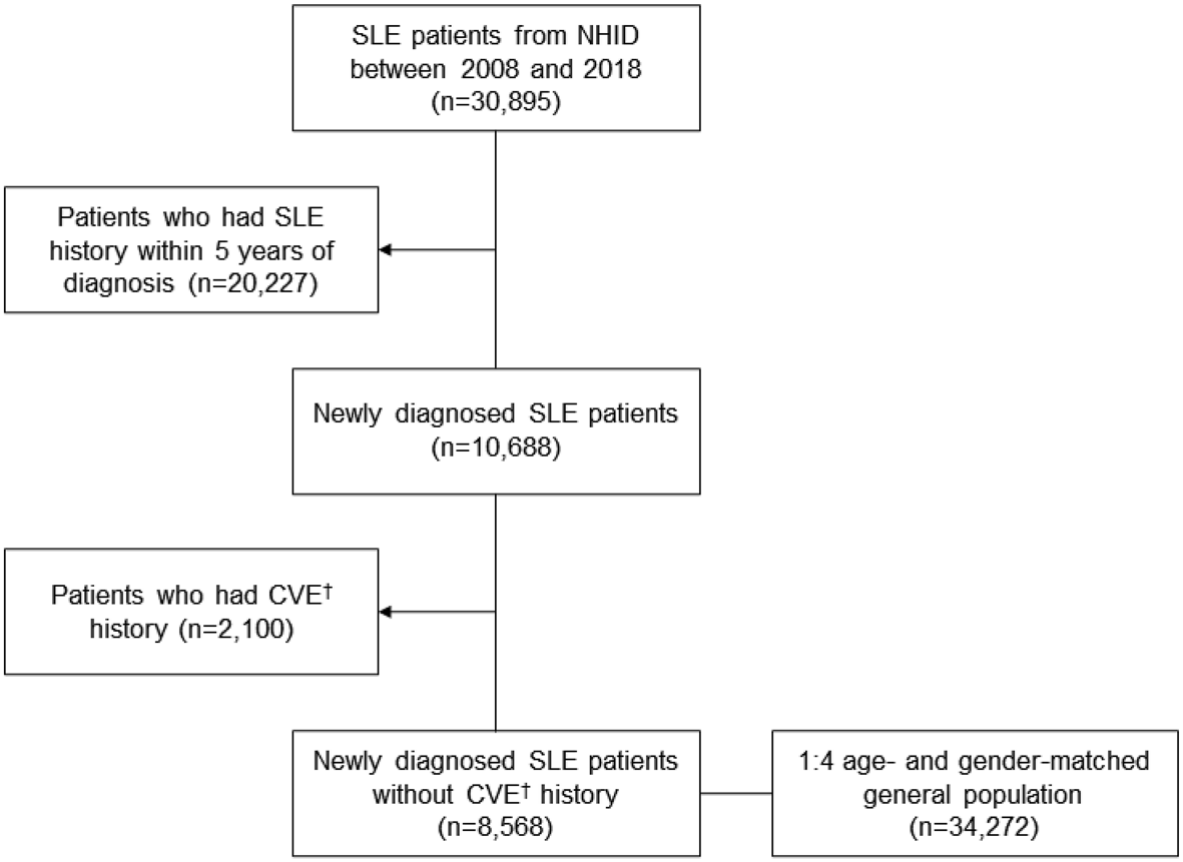
Patient selection flow. NHID, National Health Insurance service Database. CVE, Cardiovascular events. †CVE includes angina pectoris, myocardial infarction, ischemic heart disease, and cardiac arrest.

To identify incident SLE patients, individuals with a history of SLE within the 5 years prior to the index date were excluded. As a comparison group, we selected individuals from the general population who were matched by calendar year, age, and gender in a 1:4 ratio. These individuals had any medical claims recorded in the NHID between 2008 and 2018. To ensure comparability in terms of MACE outcomes, patients with a history of angina pectoris, myocardial infarction, stroke, and heart failure within 5 years before the index date were excluded from both the SLE and control groups. The selection process for incident SLE patients and controls is illustrated in Fig. 1.

### Study design and outcome

The index date was defined as the date of the first claim for SLE patients or the control group during the index period from January 2008 to December 2018. The follow-up period was defined as the time from the index date to each outcome or the end of the study (December 31, 2018). Baseline characteristics, including age, gender, income categories, comorbidities, and medication history, were assessed as covariates for all cohorts. Covariates such as comorbidities and medications were determined if they received a relevant code within three months before the index date. The primary outcome of interest was defined as MACE, encompassing MI and ischemic stroke. Secondary outcomes were defined separately as MI and ischemic stroke.

### Statistical analysis

Data are presented as the frequency (%) or mean ± SD. The risk of MACEs in age- and sex-matched cohorts was assessed by calculating the incidence rates (IRs) per 1000 person-years (PYs). IR was computed by dividing the number of incident cases by the total observational period, and a 95% confidence interval (CI) was determined. Trends in MACE incidence and its composites were compared to evaluate the overall burden of MACE over time. Using the Fine-Gray model, we estimated the cumulative incidence of MACE with time on the x-axis and cumulative incidence on the y-axis. Crude incidence rate ratios (IRRs) of MACE were calculated to measure the relative risk between SLE patients and the control group. Adjusted IRRs of MACE in SLE patients, accounting for income, comorbidities (hypertension, diabetes mellitus, and hyperlipidemia), and medication (non-steroidal anti-inflammatory drugs [NSAIDs] and glucocorticoids), were also estimated. The generalized estimating equation method was used to analyze the average effect of covariates. If medication was used for more than 180 days within a year, it was considered a time-varying covariate. Subgroup analyses were conducted by stratifying each cohort into age groups (18–39, 40–49, 50–59, and 60–79 years) and gender. IRs and IRRs of MACE and its composites were calculated for each age group and gender.

### Ethics approval

The databases extracted from NHIS could not be identified directly or through identifiers linked to the subjects; therefore, our study was exempted by the Institutional Review Board (IRB) of Hanyang University Hospital (IRB file No. HYUH 2020-05-041).

## Results

### Baseline characteristics of the study population

A total of 8568 patients diagnosed with SLE and 34,272 age- and sex-matched controls from the general population were identified from the Korean NHID for this study, maintaining a 1:4 ratio. The baseline characteristics of the study population are presented in Table 1. The average age of the population was 41.2 ± 14.4 years, with 87.8% of the participants being women. Income quartiles were divided into five groups, and it was observed that the lowest income quartile accounted for 25% of all SLE patients, whereas it represented 15% of the general population. Among the comorbidities assessed, hypertension, hyperlipidemia, and chronic kidney disease were more prevalent in SLE patients compared to the control group, whereas the prevalence of diabetes mellitus did not differ significantly between the two groups. In terms of medication usage, SLE patients were more likely to receive various medications, including NSAIDs, glucocorticoids, hydroxychloroquine, and immunosuppressive agents.

**Table 1:** Baseline characteristics of SLE patients and the general population.

Variables	SLE patients (n = 8568)	General population (n = 34,272)	*P*
Age, years	41.24 ± 14.44	41.24 ± 14.44	Matched
Sex, female	7527 (87.85)	30,108 (87.85)	Matched
Income			< 0.001
Quintile 1	2110 (24.63)	5068 (14.79)	
Quintile 2	1286 (15.01)	5072 (14.80)	
Quintile 3	1500 (17.51)	5613 (16.38)	
Quintile 4	1604 (18.72)	7066 (20.62)	
Quintile 5	1898 (22.15)	10,547 (30.77)	
Comorbidity			
Hypertension	1124 (13.12)	3097 (9.04)	< 0.001
Diabetes mellitus	250 (2.92)	1082 (3.16)	0.2538
Hyperlipidemia	630 (7.35)	2196 (6.41)	0.0016
Chronic kidney disease	201 (2.35)	84 (0.25)	< 0.001
Antiphospholipid antibody syndrome	60 (0.70)	1 (0.00)	< 0.001
Charlson comorbidity index	2.28 ± 1.41	0.63 ± 1.05	< 0.001
Medication*			
NSAIDs	5000 (58.36)	12,838 (37.46)	< 0.001
Glucocorticoids	6777 (79.10)	5977 (17.44)	< 0.001
Hydroxychloroquine	6115 (71.37)	52 (0.15)	< 0.001
Immunosuppressive agent	2785 (32.50)	113 (0.33)	< 0.001
ACE/ARB	1294 (15.1)	1937 (5.65)	
Anti-platelet agent	770 (8.99)	867 (2.53)	< 0.001
Beta blocker	547 (6.38)	695 (2.03)	< 0.001
Calcium channel blocker	1153 (13.46)	1773 (5.17)	< 0.00
Cholesterol-lowering agent	840 (9.80)	1766 (5.15)	< 0.001

Numerical quantitative data are presented as the mean ± SD and categorical data as the frequency (%). * ≥ 1 medication description for the period of 3 months before the index date.

### Increased risk of MACE in SLE patients

**Figure 2:**
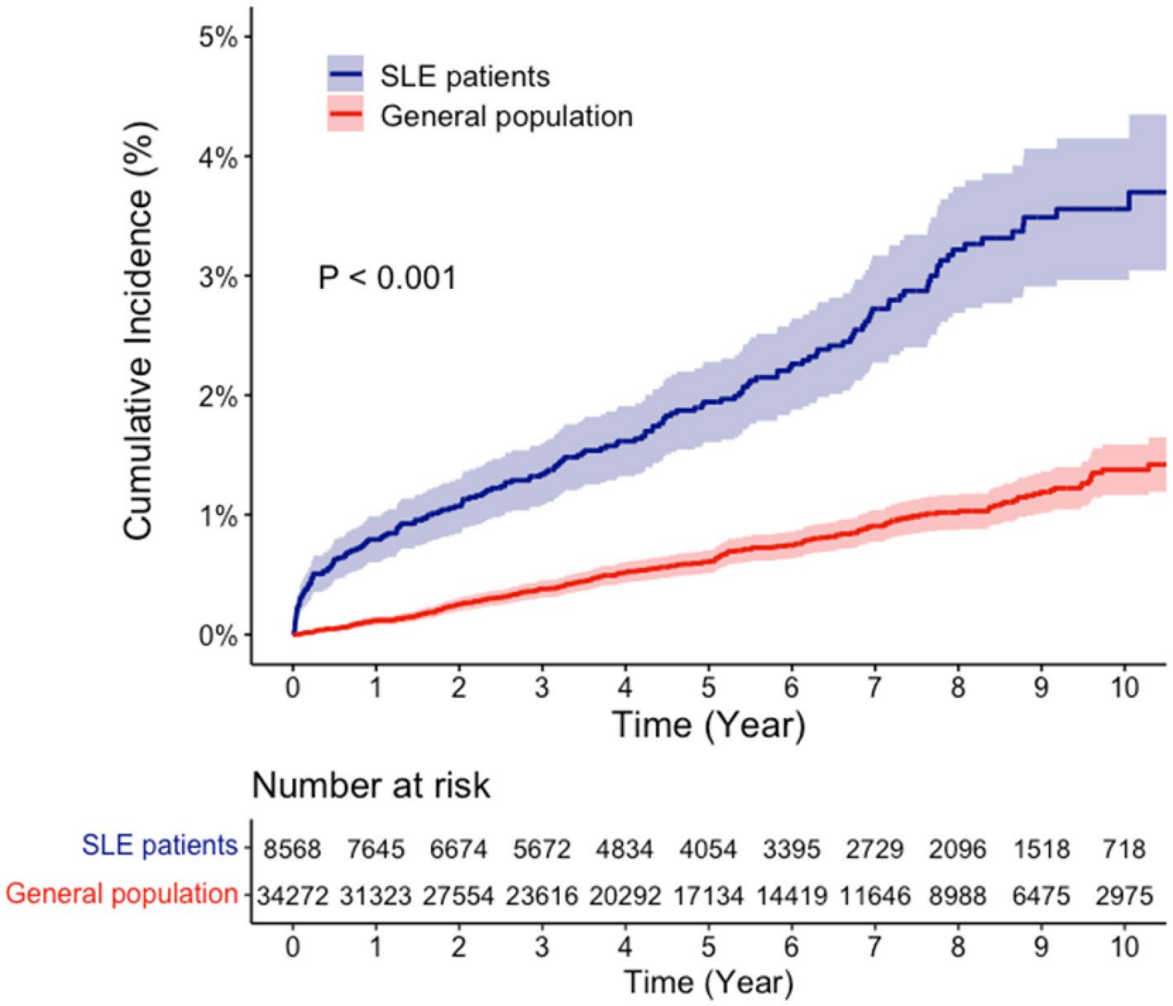
The cumulative incidence of the major adverse cardiovascular events (MACE) in patients with systemic lupus erythematosus (SLE) after diagnosis and the general population.

Table 2 presents the IRs and IRRs of MACE in SLE patients. A total of 178 MACE cases were observed during 43,579 PYs of follow-up and the crude IR for MACE in SLE patients was 4.08 per 1000 PYs. In comparison, for the general population, 237 MACE cases occurred during 181,907 PYs of follow-up, resulting in a crude IR of 1.30 per 1000 PYs. The cumulative incidence rate (CIR) of developing MACE over time, as shown in Fig. 2, was significantly higher in SLE patients than in the general population (p < 0.001). At one, five, and ten years, the CIRs of MACE in SLE patients were 0.80%, 1.94%, and 3.56%, respectively, whereas the corresponding CIRs in the general population were 0.12%, 0.61%, and 1.43%, respectively. The IRR of MACE in SLE patients was 3.14 (95% CI 2.59–3.82). After adjusting for income, comorbidities, and medication usage, the adjusted IRR of MACE was 2.40 (95% CI 1.88–3.05).

**Table 2:** The incidence rate and relative risk of MACE in SLE patients and the general population.

	Observational period (PYs)	No. of cases	Incidence rate (n/1000 PYs)	Crude IRR (95% CI)	Adjusted IRR (95% CI)*
All patients					
SLE patients	43,579	178	4.08	3.14 (2.59, 3.82)	2.40 (1.88, 3.05)
General population	181,907	237	1.30	Ref	Ref
Age (years)					
18–39					
SLE patients	23,149	51	2.20	16.16 (8.79, 29.72)	11.70 (5.95, 23.01)**
General population	95,373	13	0.14	Ref	Ref
40–49					
SLE patients	10,196	39	3.83	5.37 (3.34, 8.64)	4.00 (2.26, 7.11)
General population	42,105	30	0.71	Ref	Ref
50–59					
SLE patients	6548	44	6.72	3.27 (2.21, 4.86)	2.77 (1.72, 4.45)
General population	27,827	57	2.05	Ref	Ref
60–79					
SLE patients	3685	44	11.94	1.45 (1.03, 2.03)	1.27 (0.85, 1.90)
General population	16,602	137	8.25	Ref	Ref
Gender					
Female					
SLE patients	39,007	135	3.46	3.06 (2.45, 3.83)	2.36 (1.79, 3.12)
General population	161,862	183	1.13		Ref
Male					
SLE patients	4572	43	9.41	3.49 (1.25, 5.21)	2.50 (1.50, 4.17)
General population	20,045	54	2.69	Ref	Ref

*PY* person-year, *CI* confidence interval, *IRR* incidence rate ratio, *MACE* major adverse cardiovascular events.

*Adjusted for income, comorbidities (HTN, DM, and hyperlipidaemia), and medication (NSAIDs and glucocorticoids).

**Adjusted for income, comorbidities (HTN, and hyperlipidaemia), and medication (NSAIDs and glucocorticoids), the variable for DM was excluded from the adjusted analysis due to a poor fit.

**Figure 3:**
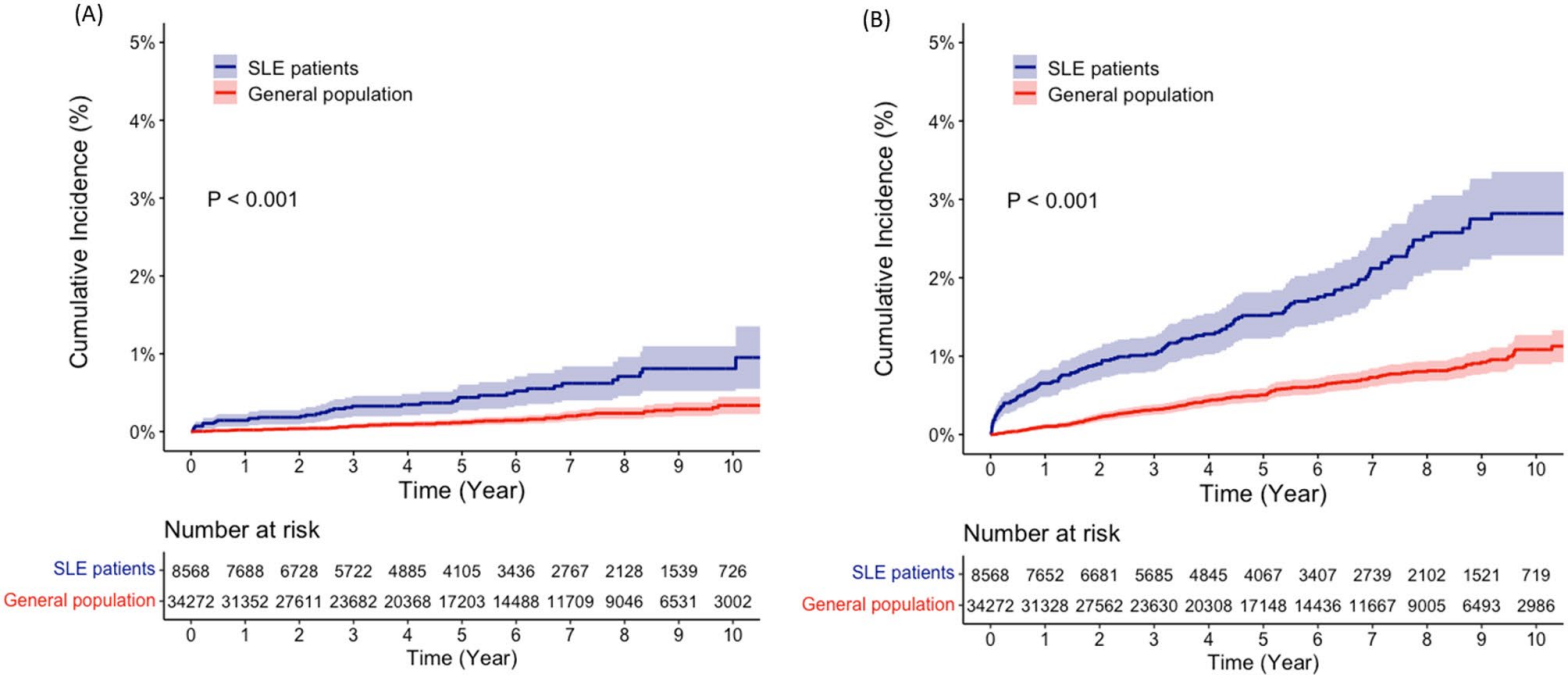
The cumulative incidence of (**A**) myocardial infarction and (**B**) ischemic stroke in patients with systemic lupus erythematosus (SLE) and the general population.

In the subgroup analysis, the adjusted IRRs for MACE were comparable between female and male SLE patients (2.36, 95% CI 1.79–3.12 and 2.50, 95% CI 1.50–4.17, respectively). When stratified by age, the crude IRs increased with age in the SLE and control groups. Among SLE patients, those aged over 60 years had the highest crude IR for MACE (11.94 per 1000 PYs), but the adjusted IRR of MACE was not significantly increased in this age group (1.27, 95% CI 0.85–1.90). The highest adjusted IRR of MACE in SLE patients compared to the general population was observed in the age group of 18–39 years (11.70, 95% CI 5.95–23.01) (Fig. 3).

### Increased risk of MI and ischemic stroke in SLE patients

Tables 3 and 4 provide information on the IRs and IRRs for each component of MACE in SLE patients. Among SLE patients, 40 cases of MI were observed during 43,977 person-years (PYs), resulting in a crude IR of 0.91 per 1000 PYs. Additionally, there were 140 cases of ischemic stroke observed over 43,663 PYs, with a crude IR of 3.21 per 1000 PYs. After adjusting for covariates, the relative risk of both MI and ischemic stroke was higher in SLE patients compared to the general population. The adjusted IRRs for MI and ischemic stroke were 2.19 (95% CI 1.30–3.68) and 2.41 (95% CI 1.84–3.15), respectively.

**Table 3:** The incidence rate and relative risk of myocardial infarction in SLE patients and the general population.

	Observational period (PYs)	No. of cases	Incidence rate (n/1000 PYs)	Crude IRR (95% CI)	Adjusted IRR (95% CI)*
All patients					
SLE patients	43,977	40	0.91	3.26 (2.15, 4.93)	2.19 (1.30, 3.68)
General population	182,482	51	0.28	Ref	Ref
Age (years)					
18–39					
SLE patients	23,277	7	0.30	7.17 (2.10, 24.51)	3.91 (1.07, 14.21)**
General population	95,416	4	0.04	Ref	Ref
40–49					
SLE patients	10,273	9	0.88	6.16 (2.19, 17.30)	3.46 (0.81, 14.21) **
General population	42,173	6	0.14	Ref	Ref
50–59					
SLE patients	6676	10	1.50	3.81 (1.62, 8.97)	3.33 (1.18, 9.41)
General population	27,980	11	0.39	Ref	Ref
60–79					
SLE patients	3752	14	3.73	2.10 (1.12, 3.97)	1.37 (0.63, 2.97)
General population	16,675	30	1.77	Ref	Ref
Gender					
Female					
SLE patients	39,324	24	0.61	2.68 (1.60, 4.48)	1.92 (1.00, 3.65)
General population	162,299	37	0.23	Ref	Ref
Male					
SLE patients	4654	16	3.44	4.96 (2.42, 10.15)	2.73 (1.08, 6.86)
General population	20,182	14	0.69	Ref	Ref

*PY* person-year, *CI* confidence interval, *IRR* incidence rate ratio, *MACE* major adverse cardiovascular events.

*Adjusted for income, comorbidities (HTN, DM, and hyperlipidaemia), and medication (NSAIDs and glucocorticoids).

**Adjusted for income, comorbidities (HTN, and hyperlipidaemia), and medication (NSAIDs and glucocorticoids), the variable for DM was excluded from the adjusted analysis due to a poor fit.

**Table 4:** The incidence rate and relative risk of ischemic stroke in SLE patients and the general population.

	Observational period (PYs)	No. of cases	Incidence rate (n/1000 PYs)	Crude IRR (95% CI)	Adjusted IRR (95% CI)*
All patients					
SLE patients	43,663	140	3.21	3.06 (2.46, 3.81)	2.41 (1.84, 3.15)
General population	182,049	191	1.05	Ref	Ref
Age (years)					
18–39					
SLE patients	23,162	44	1.90	20.13 (9.83, 41.24)	14.96 (6.75, 33.16)**
General population	95,390	9	0.09	Ref	Ref
40–49					
SLE patients	10,218	30	2.94	5.15 (3.01, 8.82)	4.21 (2.27, 7.79)
General population	42,127	24	0.57	Ref	Ref
50–59					
SLE patients	6574	34	5.17	3.07 (1.97, 4.77)	2.57 (1.51, 4.37)
General population	27,857	47	1.69	Ref	Ref
60–79					
SLE patients	3709	32	8.63	1.30 (0.88, 1.92)	1.24 (0.78, 1.97)
General population	16,675	111	6.66	Ref	Ref
Gender					
Female					
SLE patients	39,046	112	2.87	3.12 (2.44, 3.98)	2.42 (1.79, 3.28)
General population	161,973	149	0.92	Ref	Ref
Male					
SLE patients	4617	28	6.06	2.90 (1.80, 4.68)	2.36 (1.30, 4.29)
General population	20,075	42	2.09	Ref	Ref

*PY* person-year, *CI* confidence interval, *IRR* incidence rate ratio, *MACE* major adverse cardiovascular events.

*Adjusted for income, comorbidities (HTN, DM, and hyperlipidaemia), and medication (NSAIDs and glucocorticoids).

**Adjusted for income, comorbidities (HTN, and hyperlipidaemia), and medication (NSAIDs and glucocorticoids), the variable for DM was excluded from the adjusted analysis due to a poor fit.

When stratified by age and gender, the analysis revealed that SLE patients had a higher risk of MI in specific age groups. The adjusted IRRs for MI in the 18–39 and 50–59 age groups were 3.82 (95% CI 1.04–13.95) and 3.47 (95% CI 1.21–9.99), respectively. However, no statistically significant difference was found in the 40–49 and 60–79 age groups. The risk of ischemic stroke was significantly higher in SLE patients under the age of 40 years (14.96, 95% CI 6.75–33.16) compared to other age groups and there were no significant differences in the risk of MI and ischemic stroke between male and female patients.

## Discussion

Our study conducted in Korea discovered that patients who were newly diagnosed with SLE had a 2.4-fold greater risk of MACE than the general population. Additionally, our findings indicated that younger SLE patients had a significantly higher risk of MACE. The adjusted IRR of MI was higher in males compared to females, whereas no significant difference was observed in the risk of ischemic stroke between the male and female groups. When comparing the risk among different age groups, the adjusted IRR of ischemic stroke was over 14 times higher in SLE patients aged between 18 and 39 years old.

Since Urowitz et al. recognized the increased risk for MACE in SLE patients in 1976, numerous epidemiologic studies have identified elevated morbidity and mortality due to MACE^19^. We observed that the IR of MI was 0.91 per 1000 PYs with 40 cases, and the adjusted IRR for MI was 2.19. The IR and adjusted IRR for ischemic stroke were 3.21 per 1000 PYs (140 cases) and 2.41, respectively. These results, however, diverged from a previous study that reported pooled estimates of IR per 1000PYs (95% CI) of 2.81 (1.61–4.32) for MI and 4.71 (3.35–6.32) for stroke, suggesting a potential racial disparity as the contributing factor^20^. Nonetheless, our study sheds light on an intriguing aspect: the presence of a comparable risk pattern between SLE and the general population. This was demonstrated by the pooled estimates of relative risk of MI and stroke, which were determined to be 2.92 (2.45–3.48) and 2.51 (2.03–3.10), respectively^20^. Furthermore, our findings align with other Asian studies, as we observed a similar cardiovascular risk pattern when compared to research conducted in Taiwan^21^ and another study published in Korea^14^.

Recent studies showed that MACE may occur early in the course of SLE, even in patients who are newly diagnosed^22,23^. A previous study by Nived et al. reported an increased risk of MACE in newly diagnosed SLE patients^15^. It provided insights into how the duration of the disease and the age at diagnosis can potentially influence the MACE risk in SLE patients. However, they had some limitations including a small number of patients and events, resulting in significant variations in the results. Previous studies have focused on the impact of long disease duration of SLE on MACE, suggesting that an extended duration of disease influences the risk^24,25^. The significance of our study lies in its analysis of the MACE risk in the entire population over a relatively long time period, encompassing all cases. Our findings also suggested a significantly higher CIR for MACE in the first year of diagnosis, indicating MACE is not a late complication of disease. When assessing the influence of follow-up duration subsequent to the diagnosis of SLE, we observed the highest CIR ratio for MACE during the initial year after diagnosis (Supplementary figure 1). This could be interpreted as the risk for disease flare in the early stages of SLE may contribute to organ damage accrual^26^. Additionally, decreased mobility, high-dose glucocorticoids, and lupus nephritis may be associated with this trend^27^.

In general, the prevalence of traditional risk factors for MACE seems to be increased in SLE^28^. Our study showed that the frequencies of hypertension, dyslipidemia, and chronic kidney disease were increased in SLE patients. To minimize its effects, we adjusted confounding variables and observed a positive association between MACE risk and SLE. However, DM was shown to have a similar prevalence between SLE patients and the general population. Furthermore, the prevalence of DM in the 18–39 age group was significantly low; therefore, we could not include DM as an adjusting variable in this age group^29^. Nevertheless, our study showed that confounder differences for age group did not alter the observed effect estimate.

The mechanism whereby SLE contributes to MACE risk is multifactorial, and involves a complex interplay of traditional risk factors, autoimmune-mediated mechanisms, and inflammation^30^. Although the exact mechanism by which SLE increases the risk of MACE development is not fully understood, chronic inflammation might lead to the development atherosclerosis^31^. Inflammation in SLE is driven by the production of pro-inflammatory cytokines, such as tumor necrosis factor-alpha, interleukin-6, and interferon-alpha. These cytokines can promote endothelial dysfunction, platelet activation, and thrombosis, and also contribute to the development of atherosclerosis^32^. Our analyses confirm the increasing trend in the risk of MACE over time, with one exception. It is interesting that the relative risk of MACE was highest in the period immediately following SLE diagnosis. One potential explanation is that autoantibodies could be present before the clinical recognition of disease^33^. Autoantibodies are often present many years before the clinical diagnosis of SLE, and their appearance follows a predictable course, progressively accumulating before the onset of symptoms in asymptomatic patients. Therefore, additional research is required to determine whether there is an elevated cardiovascular risk during the preclinical stage or the initial phases of SLE.

One of strength in this study was that we included all cases of patients with newly diagnosed SLE in our country. This study was population based, which included large homogeneous Korean population. This may reduce the risk of selection bias and improve generalizability. Also, the NHID includes longitudinal health records, supporting researchers to follow individuals over an extended period. Despite being a retrospective study, we also estimated the cumulative incidence over more than 10 years of follow-up from the onset of SLE. By considering the occurrence of events over time, we compared the risk of MACE between SLE patients and the general population. Furthermore, traditional risk factors for MACE, such as hypertension, DM, and hyperlipidemia, are highly prevalent in SLE patients compared to the general population^34^. We collected comprehensive data on a wide range of MACE-related factors, including socio-economic status, medications, and comorbidities. By adjusting for confounding factors, we performed a detailed analysis of the factors contributing to MACE in SLE patients. Above all, we used standardized definitions for the components of MACE, which are critical outcomes with significant implications for SLE patients. This enhanced our ability to compare findings across different research studies and meta-analyses, facilitating a more comprehensive understanding of cardiovascular risk in SLE.

Our study had some limitations. This study aimed to establish an association between MACE risk and SLE, but we could not fully account for all potential confounding factors. Specifically, we could not consider certain confounding factors, both demographic and more relevantly disease-related, such as glucocorticoid dosage and SLE-related comorbidities. Second, we also acknowledge the limitation of the small sample size in outcomes. Among the SLE patients, only a small percentage developed MACE. In addition, our study was designed through incidence cases of SLE, which may have resulted in a smaller size compared to other studies. The limited occurrence of the study outcome posed challenges when conducting the multivariable analysis of risk factors. Nevertheless, the meaningfulness of our study could be derived from the fact that the HR, as seen in previous studies, was not significantly different.

This study evaluated MACE risk in newly diagnosed SLE patients, which included MI and ischemic stroke. Although the presence of comorbidities and lupus-related factors were associated with MACE, we found that SLE patients had a greater risk of MACE, especially in young patients in the 18–39 age group. These findings suggested that personalized MACE screening and modification for early SLE patients should be considered.

## Conclusion

Our large-scale nationwide cohort study provides compelling evidence to support the elevated risk of MACE in patients with SLE, particularly among the younger age group. A noteworthy finding was that the relative risk of MACE begins to increase early during the course of the disease. Consequently, it is imperative to adopt a comprehensive approach for MACE prevention in SLE, taking into consideration the age at onset and disease stage.

### Supplementary Information

Supplementary Information.

## Data Availability

The data that support the findings of this study are available from the National Health Insurance Service (NHIS) but restrictions apply to the availability of these data, which were used under license for the current study, and so are not publicly available. However, data are available from the authors upon reasonable request and with permission of the NHIS.
