# Supplementary figures and images for: Increased cardiovascular risk in Korean patients with systemic lupus erythematosus: a population-based cohort study
Source: Sci Rep. 2024 Jan 11;14:1082. doi: 10.1038/s41598-024-51546-1 (PMC10784514; doi:10.1038/s41598-024-51546-1)

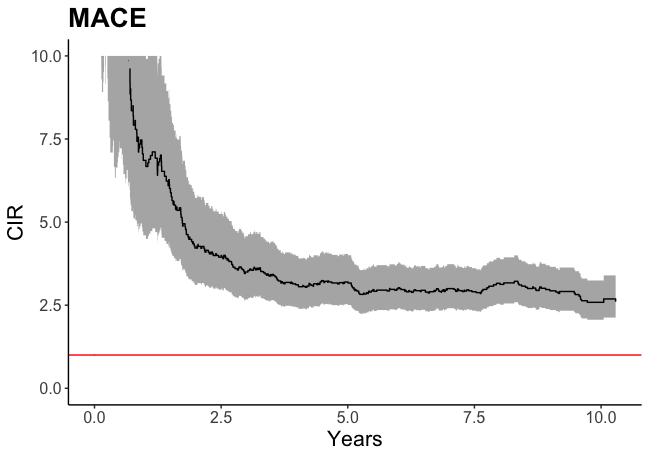

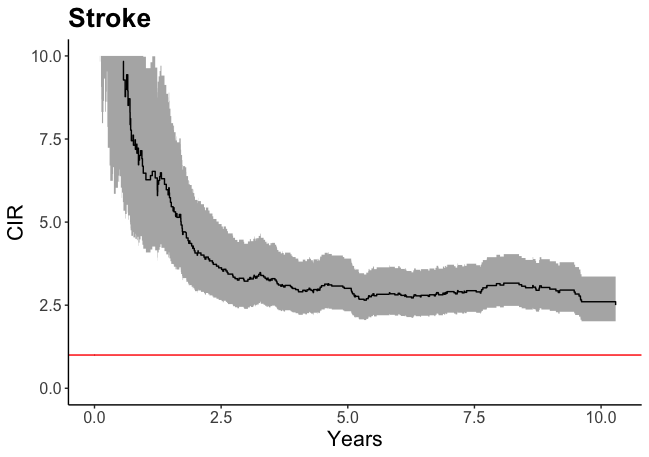

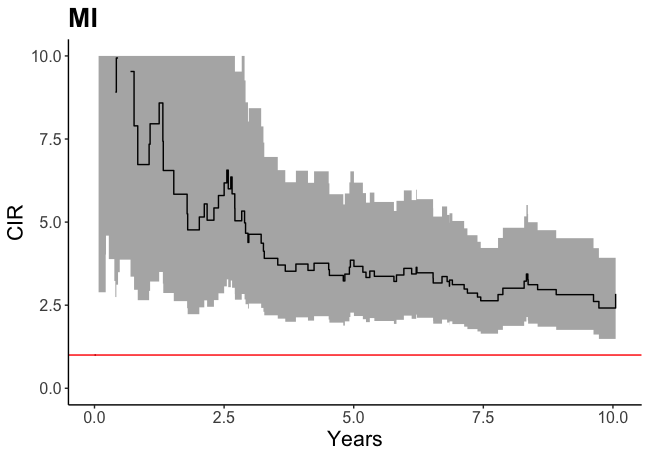


Supplementary figure 1. Cumulative Incidence Rate Ratio for MACE, MI, and stroke

Supplement: Supplementary file 1 — Supplementary Information. [file 41598_2024_51546_MOESM1_ESM.docx]
